# Health-related quality of life of people living with HIV/AIDS: the role of social inequalities and disease-related factors

**DOI:** 10.1186/s12955-021-01702-2

**Published:** 2021-02-25

**Authors:** Fikadu Tadesse Nigusso, Azwihangwisi Helen Mavhandu-Mudzusi

**Affiliations:** 1 College of Human Science, Department of Health Studies, University of South Africa (UNISA), Pretoria, South Africa; 2 Programme and Policy Section, United Nations World Food Programme, Addis Ababa, Ethiopia

**Keywords:** HIV/AIDS, Quality of life, Health-related quality of life, HRQoL, Patient-reported outcomes measurement information system (PROMIS), Social inequalities

## Abstract

**Background:**

Health-related quality of life (HRQoL) serves as a direct measure of individuals’ health, life expectancy and the impact that the utilization of health care has on quality of life. The purpose of this study is to assess the HRQoL of people living with HIV (PLHIV), and to ascertain its association with the social inequalities and clinical determinants among people living with HIV in Benishangul Gumuz Regional State, Ethiopia.

**Methods:**

A cross-sectional study was conducted between December 2016 and February 2017; 390 people at two referral hospitals and three health centers participated in the study. The Patient-Reported Outcomes Measurement Information System Global Health Scale (PROMIS Global 10) was used to measure key HRQoL domains. Global Physical Health (GPH) and Global Mental Health (GPH) summary scores were employed. GPH and GMH summary scores below 50 (the standardized mean score) were determined as poor HRQoL. Bivariate and multivariate logistic regression analyses were used to identify factors associated with GPH and GMH summary scores.

**Results:**

This study included 259 (66.4%) females and 131 (33.6%) males. The GPH summary scores ranged from 16.2 to 67.7 with a mean of 48.8 (SD = 8.9). Almost 44.6% of the study population has a GPH summary score of below 50; the GMH summary scores ranged from 28.4 to 67.6 with a mean of 50.8 (SD = 8.1). About 41.8% of the study population has a GMH summary score of below 50. Unemployment, household food insecurity and comorbidities with HIV were associated with both poor GPH and poor GMH summary scores. Age below 25 years and being a member of Christian fellowship were inversely associated with poor GPH. The least wealth index score and CD4 count below 350 cells/mL were also associated with poor GMH.

**Conclusion:**

Overall, socioeconomic inequalities and HIV-related clinical factors play an important role in improving the HRQoL of PLHIV. Many of these determinants are alterable risk factors. Appropriate strategies can improve the holistic management of chronic HIV care and maximize PLHIVs’ HRQoL. Such strategies require the adoption of comprehensive interventions, including policies and programmes that would improve the health, wellbeing and livelihood of PLHIV.

## Background

Quality of life is a broad multidimensional concept that usually includes subjective evaluations of both positive and negative aspects of life [1]; and has been recognized as a prominent measurement tool in epidemiological studies and clinical trials including the human immunodeficiency virus (HIV) or the acquired immune deficiency syndrome (AIDS) [2, 3]. When quality of life is considered in the context of health and disease, it is commonly referred to as health-related quality of life (HRQoL) to differentiate it from other aspects of quality of life [4]. HRQoL refers to how well a person functions in their life, and their perceived wellbeing in the physical, mental and social domains of health [5].

Worldwide, HIV/AIDS continues to be a major public health problem. By the end of 2018, an estimated 37.9 million people were living with HIV and AIDS globally, and 1.7 million people were newly infected by HIV [6]. To contain such an enormous number of people living with HIV and manage the disease that was once considered to be lethal, HIV care has advanced in terms of both technology and service delivery [7, 8]. Improving the overall wellbeing and life expectancy of people living with HIV (PLHIV), managing HIV/AIDS as a chronic and survivable disease, determining the social inequalities associated with HRQoL and evaluating the impact of the disease on the HRQoL of people affected by HIV/AIDS are essential to plan better social and health-care services [9, 10].

Social inequalities in HRQoL affect the treatment outcome and life expectation of people living with HIV [11–15]. Social inequality refers to differences or the unequal distribution goods based on income, wealth, gender, age, occupation, region of residence, ethnic group, marital status and so on [16, 17]. These factors all have an influence on the social position of an individual [16]. According to Kivits et al. [11], HRQoL is affected by social factors such as level of education, occupational status and net income per household. Though the magnitude of social inequalities is severe among people living with HIV in sub-Saharan Africa [18–20], social inequalities also exist globally, and have been found to affect the HRQoL of people living with HIV globally. For example, Passos et al. [12] document that in Brazil low education levels, low socioeconomic class, unemployment, sociodemographic factors (gender and age), and HIV disease-related factors such as never taking antiretroviral medication, comorbidities, HIV-related hospitalizations and a CD4 + cell count of less than 350 are associated with lower quality of life. Other researchers highlight that food insecurity is one of the major factors in social inequality that affects the quality of life of people living with HIV as it increases morbidity and reduces productivity [21–23].

HRQoL serves as a direct measure of individuals’ health, life expectancy and the impact that the utilization of health care has on quality of life [4]. A few studies have examined the HRQoL of PLHIVs in Ethiopia, and found the majority of PLHIV on chronic care have poor HRQoL and is an area of concern for national HIV/AIDS response [24–26]. Nonetheless, there is limited information available about HRQoL. There are no documented studies on the influence of social inequalities and disease-related factors on HRQoL among people living with HIV in Benishangul Gumuz Regional State. To overcome this information gap, the researchers conducted this study in Benishangul Gumuz Regional State, northwest Ethiopia, to assess the HRQoL of PLHIV and to ascertain its association with the social inequalities and clinical determinants of HRQoL in HIV-infected people.

## Methods

### Study design and population

A cross-sectional study was conducted among PLHIVs at two referral hospitals and three health centers in HIV clinics and pharmacy refill in Benishangul Gumuz Regional State, Ethiopia. These health facilities were purposely targeted as they are utilized by the majority of PLHIVs in that region. Data were collected from December 2016 to February 2017. The sample size for this study was determined using the formula for the estimation of single proportion, n = $(\frac{{\left({\varvec{z}}\right)}^{2}{\varvec{p}}\left(1-{\varvec{p}}\right)}{{{\varvec{d}}}^{2}})$, where proportion (p) is 63% (taken from the previous study in Ethiopia [26], the margin of error (d) = 5%, and there is a 95% confidence limit (Z = 1.96). By adding 10% to cater for the non-response rate, a total of 394 respondents were enrolled in the study. Respondents were allocated to the study sites proportionally to the case load at each facility. Finally, a simple random sampling technique using a sampling frame developed from the registration book of the patients was used to enroll respondents daily at each study site. The study inclusion criteria were PLHIV who (a) are on antiretroviral therapy (ART) and has follow-up consultations at the selected health facilities; (b) have resided in Benishangul Gumuz Regional State for at least 2 years; (c) have no psychiatric health problems; and (d) have signed consent to participate in the study. People under 18 years of age and critically ill patients were excluded from the study.

### Measurement of study variables

A structured questionnaire was used for face-to-face interviews with the study participants. Before data collection commenced, the study tool was piloted with a group of health care providers at the selected facilities over a period of 2 weeks to ensure clarity and consistency. The result assisted in ensuring the quality and consistency of the instrument. Data collectors were given training on the objectives of the study and how they could approach patients to gain their confidence. Interviews were conducted while participants were waiting to see ART service providers or to have prescriptions filled. Data collection forms were reviewed daily for completeness. The collected data were entered into a database and processed.

### Health-related quality of life (HRQoL)

HRQoL is the perceived physical and mental health of an individual or a group over time [1]. In this study, the Global Physical Health (GPH) and Global Mental Health (GMH) summary scores are used to measure the HRQoL. The 10-item Patient-Reported Outcomes Measurement Information System® (PROMIS®) Global Health Scale (PROMIS Global 10) was used to measure the key HRQoL domains. The PROMIS Global 10 short form consists of 10 items that assess general domains of global physical health (overall physical health, physical functioning, pain and fatigue) and global mental health (quality of life, mental health, satisfaction with social activities and emotional problems in HRQoL) [27, 28] and has largely been adapted from other frequently used “legacy” measures such as the SF-36 and EQ-5D [29, 30]. The PROMIS-10 can be used with the general population and with individuals living with chronic conditions – it has been found to be reliable and valid in diverse samples of people with chronic illnesses [27, 31, 32].

The GPH and GMH summary scores are based on PROMIS Global T-score values generated based on the principal component analysis conversion, a method that is used to summarize and group together correlated variables and confirm the presence of a single component. T-score distributions are standardized such that 50 represents the average (mean), and the standard deviation around that mean is 10 points. A high score always represents more of the concept being measured, and poor HRQoL was defined as one standard deviation or more below the PROMIS population norm (PROMIS® 2010:1). The sampling adequacy of the study was assessed by examining the Kaiser–Meyer–Olkin test (KMO test) sampling adequacy study [33]. The KMO index ranges from 0 to 1, with 0.6 suggested as the minimum value for a good factor analysis [34]. The value of KMO index was calculated as 0.841.

### Outcome measures

To assess the HRQoL of people living with HIV and ascertain its association with the social inequalities and clinical determinants, we used socioeconomic indicators such as education, income, asset possession, employment status, nutritional status, household food insecurity and sociodemographic data. Sociodemographic data included age, gender, ethnicity and marital status. Clinical determinants included CD4 count, period on ART and history of opportunistic infections. Clinical and laboratory data were collected by means of interviews and medical record reviews.

**Food insecurity** is defined as “the economic and social condition of limited or uncertain access to adequate food” [35]. The domains of anxiety about household food access, insufficient quality of food and insufficient food intake in the past 30 days were analyzed to generate a household food insecurity access scale [36].

**Asset possession** refers to a household’s possession of assets. Relevant information was elicited by asking participants a series of 13 questions about household assets and housing characteristics. A wealth index was generated using principal component analysis by combining socioeconomic variables and household characteristics [37].

**Malnutrition** is the condition that occurs when the body does not get enough nutrients [38]. This is determined based on the body mass index (BMI), which can be measured as weight in kilograms divided by the square of height in meters (kg/m^2^). Participants with a BMI of less than 18.5 kg/m^2^ were considered malnourished. Inclusion of independent variables was based on literature reviews conducted, data availability and theoretical relevance [39].

### Statistical analyses

SPSS 24.0 (IBM Corporation, Armonk, NY, USA) was used for data analysis. The GPH and GMH were analyzed based on PROMIS Global T-score values. To arrive at a bottom-line, T-score distributions are standardized such that 50 represents the average (mean), and the standard deviation around that mean is 10 points. A high score of above the PROMIS population norm of 50 represent more of the concepts measured; but a score below 50 indicates poor of the concepts being measured [40]. For this study, poor GPH and GMH (thus, poor HRQoL) is defined as one standard deviation or more below the PROMIS population norm of 50.

For data analysis and sample characterization, descriptive statistics were used, with simple frequency, measures of central tendency and variability. Binary logistic regression analysis was used to find associations between the independent variables and the outcome variable. The outcome variables were dichotomized in relation to PROMIS cut-off measures employed. All the variables showing a significant association in a bivariate analysis at p < 0.25 were entered into a stepwise backward Wald multivariate linear regression model to determine social inequalities and disease-related factors associated with the HRQoL. In the final multivariate analysis, the test was two-sided and p value < 0.05 was considered statistically significant.

## Results

### Sociodemographic and clinical characteristics

Table 1 shows sociodemographic, economic and HIV-related clinical characteristics of our analysis sample of 390 peoples living with HIV in Benishangul Gumuz Regional State, Ethiopia. This study included 259 (66.4%) females and 131 (33.6%) males, with only 98.9% response rate. The age of subjects ranged from 18 to 67 years, with a mean age of 36.1 and a standard deviation (SD) of 8.65 years; 77.2% have a Christian religious affiliation; and 91% live in urban residential areas. Ethnically, 61.5% were Amhara, 20.0% Oromo, 9.2% Agew, 3.1% Bertha and the rest were from other ethnic groups living in the region. About 50.0% were married, while 38.2% were either divorced or widowed at the time of data collection.

**Table 1:** Sociodemographic, economic and clinical characteristics of peoples living with HIV in Benishangul Gumuz Region, Ethiopia, 2020

Characteristics	Frequency (n, %)
*Sociodemographic characteristics*	
Age (in years)	
Below 25	33 (8.5)
25–35	182 (46.7)
Above 35	175 (44.9)
Gender	
Female	259 (66.4)
Male	131 (33.6)
Marital status	
Divorced/widowed	149 (38.2)
Married	195 (50.0)
Single	46 (11.8)
Religion	
Christian	301 (77.2)
Muslim	89 (22.8)
Ethnic group	
Amhara	240 (61.5)
Oromo	78 (20.0)
Agew	36 (9.2)
Berta	12 (3.1)
Others	24 (6.2)
Residence area	
Urban	355 (91.0)
Rural	35 (9.0)
*Socioeconomic characteristics*	
Education level	
Never been to school	142 (36.4)
Primary level	166 (42.6)
Secondary level	66 (16.9)
College/university level	16 (4.10
Employment status	
Unemployed	122 (31.3)
Employed	268 (68.7)
Monthly income (in Ethiopian Birr)	
< 1400	264 (67.7)
1401–2800	89 (22.8)
> 2800	37 (9.5)
Food security status	
Food insecure	296 (76)
Food secure	94 (24)
BMI score (in kg/m^2^)	
< 18.5	235 (60)
> 18.5	155 (40)
Household wealth index tertile	
1st tertile	234 (60)
2nd tertile	76(19.5)
3rd tertile	80 (20.5)
*Clinical features*	
Duration of ART initiation	
Less than 12 months	37 (9.5)
1–5 years	155 (39.7)
5–10 years	168 (43.1)
> 10 years	30 (7.7)
Recent CD4 count (in cells/mL)	
< 350	108 (27.7)
350–500	81 (20.8)
> 501	201 (51.5)
Comorbidities	
Yes	131 (33.6)
No	259 (66.4)

N, frequency in number; %, percentage

The socioeconomic characteristics of participants were as follows: 36.4% had no formal education. Most of the participants were poor as measured by the wealth index (a proxy measure for asset possession), with 60% of them in the least relative wealth tertile, 19.5% in the middle, and only 20.5% in the highest relative wealth tertile. More than a quarter of participants were unemployed (31%). A large number of the participants (67.7%) lived on a poor mean monthly income of 1,260 Ethiopian Birr (equivalent to US$45), which is below the World Bank absolute poverty threshold of US$1.90/day. The vast majority of the study participants (76%) were food insecure and (60%) had a BMI of less than 18.5 kg/m^2^.

All participants had been on ART follow-up for a mean duration of 2.49 years (SD = 0.77 years), while 7.7% of them had been on ART for more than 10 years. The mean CD4 count was 559 (SD = 319.6) cells/mL, with a range of 60 to 1,914 cells/mL. More than a quarter of them (33.6%) were frequently ill, with comorbidities in the previous three months that varied from pneumonia and tuberculosis to numerous opportunistic infections. Most of the participants were followed up at a public hospital (74%) and at a health center (26%).

### Health-related quality of life (HRQoL)

The GPH summary score ranged from 16.2 to 67.7, with a mean of 48.8 (SD = 8.9). Almost 44.6% of the study population had a GPH summary score of below 50. The GMH summary score ranged from 28.4 to 67.6, with a mean estimated at 50.8 (SD = 8.1); 41.8% of the study population had a GMH summary score of below 50.

### Factors associated with Global Physical HRQoL

Sociodemographic and economic inequalities, and clinical determinants associated with HRQoL of PLHIV were explored using bivariate logistic regression analysis and subsequent multivariable logistic regression analyses. Age, ethnic group, religious affiliation, place of residence, income, food security status, wealth index and current CD4 count have a statistical association with the GPH quality of life domain; while gender, age, marital status, education level and duration on ART were among the factors not showed statistical association with GPH in the bivariate analysis. Table 2 presents the bivariate regression analysis of GPH and GMH of the study with selected study variables.

**Table 2:** Study sample characteristics and bivariable linear regression analysis among PLHIVs in Benishangul Gumuz Region, Ethiopia, 2020

Predictors	HRQoL					
	GPH (Summary score < 50)			GMH (Summary score < 50)		
	Bivariate model			Bivariate model		
	OR	95% CI	p value	OR	95% CI	p value
*Sociodemographic characteristics*						
Gender						
Female	1.29	[0.85, 1.97]	0.23	1.38	[0.89, 2.12]	0.14
Male	1.00			1.00		
Age (in years)						
Below 25 years	0.64	[0.30, 1.35]	0.24	1.16	[0.55, 2.46]	0.70
25–35 years	0.98	[0.64, 1.49]	0.92	1.26	[0.83. 1.92]	0.28
Above 35 years	1.00			1.00		
Marital status						
Married	1.10	[0.56, 2.12]	0.78	1.62	[0.83, 3.13]	0.16
Divorced/widowed	0.60	[0.31,1.15]	0.124	0.85	[0.44, 1.65]	0.63
Single	1.00			1.00		
Ethnic group						
Amhara	1.15	[0.57, 2.33]	0.68	1.22	[0.59, 2.53]	0.59
Oromo	1.18	[1.01, 7.43]	0.031	1.29	[0.57, 2.93]	0.53
Agew	1.12	[0.44, 2.82]	0.814	1.97	[0.77, 5.08]	0.16
Others	1.00			1.00		
Religious affiliation						
Christian	0.43	[0.26, 0.71]	0.001	0.67	[0.42, 1.07]	0.097
Muslim	1.00			1.00		
Place of residence						
Urban	2.26	[1.10, 4.63]	0.026	1.24	[0.60, 2.54]	0.56
Rural	1.00			1.00		
*Socioeconomic characteristics*						
Education level						
Never been to school	1.15	[0.41, 3.23]	0.79	2.19	[0.67, 7.14]	.191
Primary level	1.34	[0.48, 3.74	0.58	2.47	[0.76, 7.98]	.130
Secondary level	1.27	[0.43, 3.81]	0.66	1.71	[0.49, 5.91]	.393
College/university level	1.00			1.00		
Employment status						
Unemployed	1.95	[1.25, 3.04]	0.003	2.18	[1.41, 3.37]	< 0.0001
Employed	1.00			1.00		
Monthly household income (in Ethiopian Birr)						
< 1400	1.88	[1.19, 2.96]	0.007	1.64	[1.03, 2.63]	0.039
1400–2400	1.41	[0.69, 2.86]	0.343	1.27	[0.61, 2.63]	0.517
> 2400	1.00			1.00		
Food security status						
Food insecure	2.5	[1.55, 4.02]	< 0.0001	7.97	[4.08, 12.5]	< 0.0001
Food secure	1.00			1.00		
BMI score (in kg/m^2^)						
< 18.5	1.29	[0.86, 1.94]	0.22	1.77	[1.16, 2.70]	0.008
> 18.5	1.00			1.00		
Wealth index tertile						
3rd tertile	0.57	[0.34, 0.96]	0.034	3.49	[3.19, 7.82]	0.007
2nd tertile	0.74	[0.39, 1.41]	0.362	0.74	[0.39, 1.37]	0.337
1st tertile	1.00			1.00		
*Clinical features*						
Duration on ART						
1 year or less	1.25	[0.61, 2.55]	0.54	1.25	[0.61, 2.54]	0.54
1–5 years	1.1	[0.71, 1.66]	0.69	1.43	[0.93, 2.18]	0.11
> 5 years	1.00			1.00		
Recent CD4 count (cells/mL)						
< 350	1.65	[1.02, 2.68]	0.042	2.42	[1.50, 3.90]	< 0.0001
350–500	0.72	[0.43, 1.22]	0.22	0.58	[0.58, 1.71	0.99
> 501	1.00			1.00		
Comorbidities						
Yes	1.65	[1.88, 2.07]	0.002	1.86	[1.22, 2.85]	0.004
No	1.00			1.00		

*HRQoL* health-related quality of life, *GPH* global physical health, *GMH* global mental health summary, *OR* odds ratio, *CI* confidence interval

In multivariate analysis, age, religious affiliation, employment status, household food security status and comorbidities have a statistically significant association with GPH. Those PLHIVs aged below 25 years was found 0.4 times less likely to be associated with poor GPH (AOR 0.4; 95% CI 0.29, 0.47; p = 0.043). Unemployed PLHIVs were found almost twice more likely to have poor GPH than employed individuals (AOR 1.87; 95% CI 1.06, 2.80; p = 0.027). In addition, food-insecure participants were twice as likely to have a poor GPH (AOR 2.31; 95% CI 1.36, 3.94; p = 0.002). Coexistence comorbidities with HIV in the last three months is found associated with high odds of being in the state of poor GPH (AOR 1.34; 95% CI 1.11, 1.62; p = 0.036).

### Factors associated with Global Mental HRQoL

In bivariate logistic regression analysis, employment status, income, household food security, BMI, wealth index, CD4 cell count and comorbidities with HIV were found to have a statistically significant association with GMH (see Table 2). However, during subsequent multivariate analysis, only marital status, employment status, household food security status, wealth index, CD4 cell count and comorbidities with HIV were remained in the final multivariate logistic regression analysis. Marital status did not show statistical significance in GMH during the final steps in backward Wald multivariate analysis (see Table 3).

**Table 3:** Global Physical and Mental HRQoL summary scores and multivariable linear regression analysis with selected study sample characteristics in Benishangul Gumuz Region, Ethiopia, 2020

Variables	Multivariate analysis		
	AOR	95% CI	p value
*GPH*			
Age below 25 years	0.40	[0.29, 0.47]	0.043
Married	1.04	[0.49, 2.13]	0.920
Divorced/widowed	0.52	[0.25, 1.07]	0.074
Amhara	1.01	[0.17, 1.02]	0.390
Oromo	1.16	[1.32, 7.09]	0.400
Agew	1.08	[0.39, 2.94]	0.830
Christian	0.39	[0.23, 0.69]	0.001
Unemployed	1.87	[1.06, 2.80]	0.027
Food-insecure	2.31	[1.36, 3.94]	0.002
Comorbidities	1.34	[1.11, 1.62]	0.036
*GMH*			
Married	1.31	[0.62, 2.80]	0.482
Divorced/widowed	0.74	[0.35, 1.53]	0.425
Unemployed	2.65	[1.04, 2.65]	0.035
Food-insecure	6.43	[3.22, 9.82]	< 0.0001
3rd tertile wealth index	1.77	[1.21, 3.38]	0.036
2nd tertile wealth index	0.82	[0.41, 1.67]	0.587
CD4 count below 350 cells/mL	1.91	[1.14, 3.21]	0.014
CD4 count 350–500 cells/mL	1.04	[0.58, 1.89]	0.090
Comorbidities	1.52	[1.62, 3.68]	0.042

*AOR* adjusted odds ratio

Unemployed PLHIVs were 2.65 times more likely to have poor GMH than those who had jobs (AOR 2.65; 95% CI 1.04, 2.65; p = 0.035). Food-insecure people living with HIV were around 6.43 times more likely to have poor GMH than their food-secure counterparts (AOR 6.43; 95% CI 3.22, 9.82; p < 0.0001). Participants in the third tertile of the wealth index (those with the least wealth) were almost twice as likely to have poor GMH than those in the second tertile of the wealth index (AOR 1.77; 95% CI 1.21; 3.38; p = 0.036). It was also shown that those PLHIVs who reported to have a current CD4 count below 350 cells/mL were 1.91 times likely to have poor GMH than those whose CD4 count was above 350 cells/mL (AOR 1.91; 95% CI 1.14, 3.21; p = 0.014). Likewise, those PLHIVs who were sick with HIV/AIDS-related comorbidities were found 1.52 times more likely to have poor GMH than those without the conditions (AOR 1.52; 95% CI 1.62, 3.68; p = 0.042).

## Discussions

Our study used the Patient-Reported Outcomes Measurement Information System® (PROMIS®) instrument to assess the HRQoL among PLHIV in Benishangul Gumuz Regional State, Ethiopia, and attempted to demonstrate the importance of factors in the sociodemographic, economic and clinical variables to quality of life. Overall, PLHIVs in this study have shown to have poor GPH (mean = 48.8, SD = 8.9) in comparison to GMH (mean = 50.8, SD = 8.1). Affiliation to the Christian religion group, unemployment, household food insecurity and comorbidities with HIV were identified as the most important socioeconomic and clinical determinant factors of poor global physical health related quality of life among PLHIVs. Participants aged younger than 25 years were found to have good GPH summary scores in comparison to participants older than 25 years of age.

Our findings correspond with the reports of Abera et al. [41] that the mean values of GPH scores decline with age. In Brazil [42], PLHIVs aged over 25 years were strongly associated with poor GPH summary scores. Another study in Brazil [43] has shown that a young age (below 25 years) predicts higher global physical HRQoL. Likewise, evidence from a meta-analysis of social and demographical determinants of quality of life in people who live with HIV/AIDS has shown that those who are under 35 years of age have a positive and direct association with better physical HRQoL [13]. The higher levels of global physical health among the younger population shows higher levels of physical strength, energy and exercise.

Several studies have shown that certain sociodemographic factors, namely religious affiliation, is associated with quality of life among PLHIVs [14, 44, 45]. In our study, Christian followers have better global physical health-related quality of life. Although the role of religious differences was not well understood, differences in socioeconomic factors: specifically lifestyles and religious beliefs, play a significant role in affecting physical HRQoL [46, 47]. The differences in religious affiliations have also been shown to affect the HRQoL of PLHIVs on combination ART in different setups, including in Addis Ababa, Ethiopia [14], Brazil [48], and China [44]. It is reported that religion is traditionally strongly tied with HRQoL and very important for PLHIVs [49], nevertheless the question why PLHIVs affiliated to particular religious group have lower GPH scores needs to be answered by future studies.

Low levels of socioeconomic status, which are characterized by unemployment and food insecurity, are strongly associated with poor GPH score domains of HRQoL. Our study shows that unemployed PLHIVs are nearly twice more likely to have poor GPH. Likewise, it has been shown that unemployment leads to poor HRQoL among PLHIV in both developed and developing countries [13, 18, 50]. A study in Ethiopia has found that unemployed PLHIVs are twice as likely to have poor physical HRQoL than employed PLHIVs [24]. Reports in different setups have shown that food insecurity is a major contributor to poor HRQoL. Our findings reveal that food insecure PLHIVs were 2.31 times more likely to have poor GPH summary scores. Research in Uganda has also shown that PLHIV from households that suffer severe food insecurity have the lowest mean physical health status scores [21, 22]. Equally, a longitudinal community-based research study in Canada [51] has found that food insecurity is consistently associated with poor physical health scores. It is evident that the relationship between food insecurity and HIV is complex, and food insecurity may impact both indirectly and directly on physical and functional HRQoL. For example, both food insecurity and HIV infection result in a gradual decline in immunity, which may in turn result in lower GPH summary scores. Thus, food assistance for food-insecure PLHIV can significantly increase the physical health scores and is reported to positively affect the physical HRQoL of PLHIVs in Uganda [52].

Unemployment and food insecurity have also been found to be predictors of poor global mental HRQoL in our study. Like previously reported studies [12, 15, 53, 54], our study indicates that unemployed PLHIV have poor GMH scores. Studies in Ethiopia have established that there is a strong link between unemployment and HRQoL [55, 56]. For people living with HIV, unemployment is an interactional and reinforcing process for poor mental health [54]; it has an adverse effect on psychological wellbeing and the HRQoL of an individual, resulting in financial limitations and a poor standard of living [15]. Our study shows that food-insecure households are nearly seven times more likely to have poor global mental health scores than their counterparts. This confirms the finding of studies in South Indian that show that food-insecure PLHIVs have the lowest mental health score [57], and food insecurity affects mental HRQoL as it leads to mental health problems such as major depression [51, 58, 59]. Depression accelerates the disease progression of HIV to AIDS, which can affect the appetite and food security status of PLHIV, and eventually results in lower quality of life [58]. Mental HRQoL measures wellbeing and reflects individuals’ assessments of the impact of their mental health on their social participation in their current environment. A study conducted in Uganda has found that food assistance to food-insecure PLHIV does not lead to an increase in their mental health score [52].

This study also shows that the third tertile on the wealth index (or the poorest PLHIVs in terms of asset possession) has a strong association with GMH, which is consistent with reports by Weldsilase et al. [60] and Abebe [61] that least-wealth status is associated with poor overall HRQoL. From these reports it is very evident that a lack of assets affects the health of PLHIVs as manifested psychologically in poor mental HRQoL. Least wealth may also be an indication of socioeconomic inequalities resulting from unemployment, lost work opportunities as a result of gender disparities, which leads to poor access to health care and poor overall health. Nevertheless, it is not clear whether a poor position on the wealth index can be contributed to HIV infection as no further data related to asset possession have been collected.

It has been found that there is a strong correlation between HIV-related clinical outcomes, such as CD4 < 350 cells/mL, and poor GMH summary scores. Comorbidities with HIV are strongly associated with both poor GPH and poor GMH summary scores. Like our study, studies in Nigeria [62], Southern Brazil [63], Bangladesh [64] and USA [65] have shown that a CD4 + cell count of less than 350 cells/mL is associated with poorer HRQoL. The lower the CD4 count, the more susceptible PLHIVs are to infection and mental stress. A higher CD4 + cell count reflects an improved health status as the body is capable of protecting itself from comorbidities, which in turn leads to better HRQoL. Yet, there are also studies that show no association between CD4 + cell counts and HRQoL among people with HIV in an Irish cohort [15], among HIV-infected adolescent and young adult women in the USA [65] and in Taiwanese PLHIV [66]. The disagreement may be attributed to differences in study populations and differences in individuals’ perceptions of their health. Consistent with the report of the study in Ethiopia [67], it has been found that comorbidities with HIV is strongly associated with both GPH and GMH, resulting in poorer HRQoL. Several study reports agree with these findings [68, 69] that additional comorbidities with HIV exposes PLHIV to a double burden of illnesses that could significantly compromise their quality of life. The presence of additional comorbidities directly affects the general functioning and wellbeing of PLHIV and thus mediates poor HRQoL.

### Limitations

The limitations of this study include its cross-sectional nature, which may preclude conclusions on causality. Hence the exact direction of the associations between predictor variables and HRQoL cannot be established. Recruitment of participants from the outpatient clinics and exclusion of hospitalized PLHIV may have altered the HRQoL findings. Finally, other confounding factors such viral load, clinical stage, alcohol use and smoking were not considered in the questionnaires. For further exploration, we would take these factors into consideration.

## Conclusions

The study reveals that socioeconomic inequalities are important determinants of improved HRQoL among PLHIVs. Factors such as being older than 25 years, unemployment, household food insecurity and comorbidities with HIV are significantly associated with poor physical HRQoL. Conversely a Christian religious affiliation is associated with higher global physical health-related quality of life. Unemployment, food insecurity, least wealth index score, recent CD4 count below 350 cells/mL and comorbidities with HIV are associated with poor global mental HRQoL. Many of the predictors are alterable risk factors. Appropriate strategies can improve the holistic management of chronic illness and maximize PLHIVs’ HRQoL. Improving the HRQoL of PLHIVs requires comprehensive intervention strategies, including policies and programmes that will improve the health, wellbeing and livelihood of PLHIVs. For instance, age-specific physical health strengthening activities for adults over 25 years of age improve physical HRQoL. Faith-based behavioral and physical intervention strategies improve religious determinants of poor physical HRQoL, and tackling the issue of unemployment and food insecurity improves the physical HRQoL [13, 45, 51]. In addition, addressing social inequality brought about by unemployment, food insecurity and poor asset possession can enhance mental HRQoL. Improving GMH summary scores emphasize the need for interventions to boost the immunity and CD4 count of PLHIV, and to prevent and treat comorbidities timeously.

## Data Availability

Please contact author for data requests.

## Abbreviations

AIDS: Acquired Immune Deficiency Syndrome
AOR: Adjusted odds ratio
ART: Antiretroviral therapy
BMI: Body mass index
CI: Confidence interval
HIV: Human immunodeficiency virus
HRQoL: Health-related quality of life
GMH: Global mental health
OR: Odds ratio
GPH: Global physical health
PLHIV: People living with HIV and AIDS
QoL: Quality of life
